# Concave Pattern of a Maximal Expiratory Flow-Volume Curve: A Sign of Airflow Limitation in Adult Bronchial Asthma

**DOI:** 10.1155/2012/797495

**Published:** 2012-11-27

**Authors:** Akihiko Ohwada, Kazuhisa Takahashi

**Affiliations:** ^1^Ohwada Clinic, 4-7-13 Minamiyawata, Ichikawa, Chiba 272-0023, Japan; ^2^Department of Respiratory Medicine, Juntendo University School of Medicine, Tokyo 113-8421, Japan

## Abstract

*Background*. In patients with bronchial asthma, spirometry could identify the airflow limitation of small airways by evaluating the concave shape of the maximal expiratory flow-volume (MEFV) curve. As the concave shape of the MEFV curve is not well documented, we reevaluated the importance of this curve in adult asthmatic patients. *Methods*. We evaluated spirometric parameters, the MEFV curve, and its concave shape (scoop between the peak and endpoint of expiration) in 27 nonsmoking asthmatic patients with physician-confirmed wheeze and positive bronchial reversibility after a short-acting **β**2-agonist inhalation. We also calculated angle **β** and shape factors (SF_25%_ and SF_50%_) to quantitate the curvilinearity of the MEFV curve. *Results*. The MEFV curve was concave in all patients. Along with improvements in standard spirometric parameters, curvilinear parameters, angle **β**, SF_25%_, and SF_50%_ were significantly improved after bronchodilator inhalation. There were significant correlations between improvements in angle **β**, and FEF_50%_, and FEF_25-75%_, and between improvements in SF_25%_, and SF_50%_, and FEF_75%_. *Conclusions*. The bronchodilator greatly affected the concave shape of the MEFV curve, correlating with spirometric parameters of small airway obstructions (FEF_50%_, FEF_75%_, and FEF_25-75%_). Thus, the concave shape of the MEFV curve is an important indicator of airflow limitation in adult asthmatic patients.

## 1. Introduction

Evaluation of airflow limitation is crucial for diagnosis of bronchial asthma and chronic obstructive pulmonary diseases. Spirometry is a simple but important procedure to detect airflow limitation. Reductions of forced expiratory volume in 1 s (FEV_1_), FEV_1_/forced vital capacity (FVC) ratio, and peak expiratory flow (PEF) are proven signs [1].

In the 1970s and 80s, analysis of the configuration of maximal expiratory flow-volume (MEFV) curve concluded that the concave shape of the MEFV curve reflects the presence of small airway obstructions. Kraan et al. analyzed the changes in the MEFV curve in patients with bronchial asthma after treatment with inhaled steroids using indices of curvilinearity of the MEFV curve, shape factors (SFs), and slope ratio (SR) [2]. They revealed that the shape of the curve became less bowed toward the volume axis after inhaled corticosteroids and concluded that such a change in the MEFV curve reflects a decrease in the inhomogeneous distribution of airflow narrowing. Kapp et al. defined a new parameter, angle *β*, to characterize the shape of the MEFV curve and revealed that patients with asthma, chronic bronchitis, dyspnea, and wheeze had significantly lower *β* angles than healthy individuals [3].

In 2005, the American Thoracic Society (ATS)/European Respiratory Society (ERS) task force noted that the earliest changes associated with airflow obstruction in small airways are thought to be slowing in the terminal portion of the spirogram, even when the initial part of the spirogram is barely affected. This slowing of expiratory flow is most obviously reflected in the concave shape on the MEFV curve. It is reflected quantitatively in a reduced force expiratory flow (FEF) at 75% of FVC expired (FEF_75%_) or FEF between 25 and 75% of VC (FEF_25–75%_) [4]. However, abnormalities in these midrange flow measurements during forced exhalation are not specific for some cases of small airway diseases. It is thus still unclear whether the concave shape of the MEFV curve is an indicator of airflow limitation.

In this study, we analyzed the shape of the MEFV curve in patients with bronchial asthma with both physician-confirmed wheeze and positive bronchial reversibility and reevaluated the importance of the concave shape of the curve in diagnosis of bronchial asthma.

## 2. Methods

Subjects with physician-confirmed wheeze auscultated at their initial visit were enrolled from among nonsmoking asthmatic patients who complained of cough and dyspnea. Patients with obvious acute respiratory infection were excluded. None of the patients had taken inhaled steroids or *β*2 agonists for at least 3 months before the visit.

Spirometry was performed with a Microspiro HI-801 (Chest Inc., Japan) following the instructions in the ATS/ERS statements, and the highest FVC, FEV_1_, FEV_1_/FVC ratio, and PEF values of the technically acceptable recordings were taken (4,5). FEF at 25%, 50%, and 75% of FVC expired (FEF_75%_, FEF_50%_, and FEF_25%_, resp.); FEF_25–75%_ were measured from the recording that had the largest sum of FEV_1_ and FVC [4, 5]. These parameters are presented as absolute values and percentiles of the predicted values (% predicted). The spirometric reference values used have been reported by the Japanese Respiratory Society for FVC, FEV_1_, FEV_1_/FVC ratio, FEF_75%_, and FEF_50%_ [6] and elsewhere for PEF [7], FEF_25–75%_ [7], and FEF_75%_ [8].

Reversibility of airflow limitation was evaluated with FEV_1_ at 15 min after the inhalation of salbutamol 200 *μ*g using a spacer following ATS/ERS standardization [5]. Bronchial reversibility is defined as a SABA-induced increase in FEV_1_ of ≥0.20 L and ≥12% of baseline [1].

**Figure 1:**
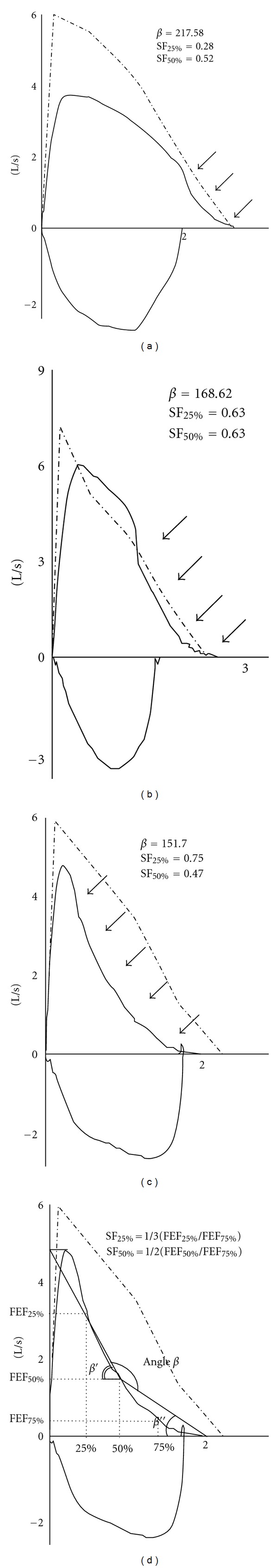
(a–c) Representative cases of various patterns of concave maximal expiratory flow-volume curves in the subjects analyzed in this study. Arrows indicate the concave region of the curve. (d) Calculation of angle *β* and shape factors (SF_25%_ and SF_50%_) from standard spirometric parameters.

Several ways to quantify the shape of the MEFV curve have been proposed. To characterize the shape of the MEFV curve using angle *β* the residual volume (RV) point of the MEFV curve was joined to the flow point at midvolume and then the flow point at midvolume to a point at the level of the total lung capacity (TLC) at the height of the peak flow above the *x*-axis. Although angle *β* could obtain graphically by tracing angle *β* as in Figure 1(d), Kapp et al. validated the use of angle *β* with the following equation: *β* = 180° − *β*′ + *β* ^″^ = 180° − tan^−1^⁡ (PEF − FEF_50%_/0.5 × FVC) + tan^−1^⁡(FEF_50%_/0.5 × FVC) (all tan^−1^⁡  values were calculated in degrees) to obtain results similar to manual tracing [3]. Hence, we calculated the angle from obtained spirometric parameters. Values of *β* < 180° correspond to the concavity of MEFV [3].

Shape factors (SFs) were other indices of curvilinearity of the MEFV curve. SF_50%_ and SF_25%_ were obtained from the respective equations: 1/2 × FEF_50%_/FEF_75%_ and 1/3 × FEF_25%_/FEF_75%_ [2]. To calculate SFs, % predicted values were applied to FEF_75%_, FEF_50%_, and FEF_25%_. A value of SF_50%_ or SF_25%_ larger than 1 indicated a concave pattern of the MEFV curve. Percent change (from baseline) was also calculated for the spirometric parameters other than FEV_1_, angle *β*, SF_25%_, and SF_50%_. All patients provided an informed consent and the study was approved by the institutional review board.

### 2.1. Statistical Analysis

Data are presented as means ± SD. Baseline spirometric parameters were compared to those after SABA inhalation by paired *t* test. Correlation coefficients for angle *β*, SF_25%_, and SF_50%_ with other spirometric parameters were calculated for % change by the non-parametric Spearman method using Graphpad Prism (Graphpad Software, Inc., San Diego, CA). *P* < 0.05 was considered to indicate a significant difference.

## 3. Results

Among 1020 nonsmoking asthmatic patients over a 20-month period, 51 (5.2%) had physician-confirmed wheeze by auscultation at their first visit. Consecutively, we evaluated bronchial reversibility in these 51 patients. Of these, 27 (51%), aged 18–68 years had bronchial reversibility. All 27 included subjects were Japanese (10 males and 17 females) and had symptoms of cough and dyspnea at their first visit. Eight patients had a past history of childhood bronchial asthma, 5 had been diagnosed adult-onset asthma prior to their visits, and 8 had a family history of asthma. Fifteen of the patients had allergic rhinitis, allergic eczema, or were positive on an allergic blood test.

The baseline values of the spirometric parameters were shown in Table 1.

The concave shape of the MEFV curve was defined as a curve with a scoop between the peak of the curve and the endpoint of expiration (Figure 1). All patients had a concave flow-volume curve. Two (7.4%) of the 27 patients had normal values for FEV_1_ (≥80% of predicted value), FEV_1_/FVC ratio (≥70%), and PEF (≥80%).

The baseline angle *β* was 172 ± 21°. Twenty (74%) of the 27 patients had angle *β* < 180°, indicating the concavity of the MEFV curve. The baseline values of SF_25%_ and SF_50%_ were 0.62 ± 0.34 and 0.61 ± 0.19, respectively. SF_25%_ and SF_50%_ values <1 were found in three (11%) and two (7.4%) patients, respectively.

The change in FEV_1_ from baseline after SABA inhalation was in Table 1. 0.61 ± 0.56 L (range: 0.20–2.67 L), with 25.4 ± 25.0% change (12.4–127.7%).

The values of the spirometric parameters after SABA inhalation were demonstrated in Table 1. All values after SABA inhalation were significantly higher than baseline values. The values of angle *β*, SF_25%_, and SF_50%_ after SABA inhalation were 188 ± 20°, 0.19 ± 0.07, 0.25 ± 0.13, respectively (Table 1). Angle *β* after SABA inhalation was increased in all patients except for one (data not shown). Both SF_25%_ and SF_50%_ were decreased after SABA inhalation in all but one. These changes indicated that the concavity of the MEFV curve improved after bronchodilating treatment.

To explore the factors that influence the changes in angle *β*, SF_25%_, and SF_50%_ after SABA inhalation, correlations were examined between their % changes and the % changes of the standard spirometric parameters (Table 2).

Improvement in angle *β* was significantly correlated with improvements in FEF_50%_ (*r* = 0.71, *P* < 0.0001) and FEF_25–75%_ (*r* = 0.57, *P* = 0.0017). Improvements (decrease in values) in SF_25%_ and SF_50%_ were strongly linked with each other (*r* = 0.9, *P* < 0.0001) and significantly correlated with improvement in FEF_75%_ (*r* = 0.53, *P* = 0.0046 for SF_25%_; *r* = 0.54, *P* = 0.0041 for SF_50%_) after SABA inhalation.

## 4. Discussion

The prevalence of current wheeze in asthmatic children varied among countries ranged from 0.8% to 32.6% in the 13-14-year olds and ranged from 2.4% to 37.6% in the 6-7-years olds [9]. The prevalence of patient-recognized wheeze in adult asthmatic subjects would also vary widely and be reported as 5.4% in Japan; that rate was consistent with our observation [10]. Moreover, positive bronchial reversibility is thought to be dependent on the baseline FEV_1_ values. Yancey and Ortega showed that patients with a baseline FEV_1_ of 40% to <50% predicted at screening had a mean reversibility of 42%, and those with a baseline FEV_1_ of 90% to <100% predicted had a mean reversibility of 18%, suggesting that a lower baseline lung function results in a higher reversibility [11]. In our previous study, we randomly enrolled 45 patients with bronchial asthma with a mean FEV_1_ value of 88.96% predicted in outpatient clinic and found that 9 (20%) of them were positive for bronchial reversibility [12]. In this study, the positive rate of bronchial reversibility was higher (51%) than that expected for patients with physician-confirmed wheeze (mean baseline FEV_1_; 72.9% predicted). Although patients with bronchial asthma are heterogeneous in respect to bronchial responsiveness to bronchodilator treatment [11], our result indicates that the patients with wheeze are well responders for bronchodilators. Hence, we chose in this study only asthmatic patients with both physician-confirmed wheeze and positive bronchial reversibility to reduce the divergence of the SABA response. Thus, our cohort was small in this outpatient clinical setting. We recognized the presence of some limitations in this study. One was small numbers of subjects. Another was whether our selected patients group belonged to peculiar or ordinary phenotype of adult asthma. Interestingly recent analysis of the Severe Asthma Research Program (SARP) revealed that severe asthma was peculiar to be characterized by abnormal lung function that is responsive to bronchodilators [13] and prominent air trapping (detected as increased RV/TLC ratio) over the entire range of airflow obstruction severity and that nonsevere asthmatic patients did not exhibit significant air trapping even at the more severe stages of airflow limitation expressed as FEV_1_/FVC ratio [14]. 

Mead developed the slope ratio (SR), defined as tangent slope (d$\dot{V}$/dV) divided by the chord $\dot{V}$/(FVC-V), as an index of the curvilinearity of the MEFV curve [15]. However, special values of FEF are needed, for example, at 87.5%, 62.5%, 37.5%, and 12.5% of FVC expired, to obtain SRs at 75%, 50%, and 25%, or the values need to be directly measured from the plotted MEFV curve [15, 16]. As it is impractical to use SRs in a standard pulmonary function test, we used angle *β*, SF_25%_, and SF_50%_ to quantify the concave pattern of the MEFV curve.

The concave shape of the MEFV curve was confirmed in 74% of the patients based on the baseline angle *β* (<180°), in 11% based on baseline SF_25%_ (<1.0), and in 7% based on baseline SF_50%_ (<1.0). One reason of rather poor concordance of these three indexes with curve shape might be due to inappropriate threshold values to classify the curve. For angle *β*, Kapp et al. demonstrated that the values for adult male and female individuals with no impairment of FEV_1_  were 190.5 ± 17.7°, 198.8 ± 17.9°, respectively [3]. Normal value of angle *β* is probably larger than 180° and is influenced with age and gender [3]. For SFs, there is no additional study found. In our study, both SF_25%_ and SF_50%_ were significantly improved along with angle *β* and other standard spirometric parameters after SABA inhalation (Table 1). This finding indicates that the values of SFs also respond to the effects of the bronchodilator. Hence we are unable to easily discard SF indexes due to poor discriminatory power in this small-scaled evaluation. Further analysis of concordance/disagreement of these three indexes with or without regard to the FEF values should be necessary. It must also apply to evaluate with the spirometric values after bronchodilating treatment.

Next, we evaluated which spirometric parameters were related to the improvements in angle *β*, SF_25%_, and SF_50%_ after SABA inhalation by analyzing correlation coefficients (Table 2). We revealed that % changes from baseline values of angle *β*, SF_25%_, and SF_50%_ were related to % changes of FEF_50%,_ FEF_75%_ and FEF_25–75%_, the parameters of airflow limitation in small airways [4, 17]. Thus, we considered that the concave shape of the MEFV curve reflected the airflow limitation in small airways.

A concave flow-volume curve has been described in a few studies of childhood asthma [18, 19]. It was suggested that the severity of asthma in school-aged children could be predicted at the first visit based on a concave flow-volume curve and the past frequency of symptoms [18]. Ethnic differences may also play a role in pulmonary function in child asthma. Hispanic girls with asthma have a larger flow deficit than non-Hispanic girls and have larger reductions in FEF_75%_, FEV_1_, and PEF [19]. Further investigations are needed to evaluate ethnic differences in the concave pattern of the MEFV curve in adult patients with bronchial asthma. Recently it was demonstrated that children with wheezing disorders had lower angle *β* than healthy children [20]. 

After several years of debate surrounding this topic, the contribution of small airway abnormalities in asthma pathobiology remains mainly unanswered. It is likely that a combination of techniques, including lung function tests such as evaluation of lung volumes through spirometry and/or the single-breath nitrogen washout, possibly associated with imaging, could be the most suitable approach. Once defined and accepted, these techniques would have to be used in a properly designed clinical study aimed at assessing the impact of treatments targeting the distal lung and possibly at establishing a correlation between the modification of distal lung parameters and improvement in the clinical status of the patient [21, 22].

In our analysis, angle *β* proposed by Kapp et al. reflected the configuration of the MEFV curve. Based on our definition of the concave shape, direct observation of the curve is simple. Even a patient with the curve seen in Figure 1(a) has wheeze and positive bronchial reversibility. Of note, two patients (7.4%) with wheeze and positive bronchial reversibility had normal values of FEV_1_, FEV_1_/FVC, and PEF, but showed a concave shape in their MEFV curves.

Here, we emphasize the importance of the shape of the MEFV curve as a simple indicator of airflow limitation, even in patients with normal values of FEV_1_, PEF, and FEV_1_/FVC.

## Figures and Tables

**Table 1:** Spirometric parameters in patients with wheeze and bronchial reversibility.

	Baseline		After SABA inhalation		*P *
	Absolute	% predict	Absolute	% predict	
FCV (L)	3.18 ± 1.05	84.4 ± 18.0	3.51 ± 1.05	93.0 ± 15.6	0.0003
FEV_1_/FCV (%)	70.7 ± 10.2		78.4 ± 10.4		<0.0001
FEV_1_ (L)	2.24 ± 0.74	72.9 ± 18.1	2.76 ± 0.92	89.1 ± 18.1	<0.0001
PEF (L/s)	4.90 ± 1.88	59.6 ± 16.5	5.64 ± 2.06	68.2 ± 17.1	0.0022
FEF_25–75%_ (L/s)	1.85 ± 0.92	48.1 ± 23.7	2.66 ± 1.38	67.4 ± 29.7	<0.0001
FEF_25%_ (L/s)	3.82 ± 1.52	63.0 ± 22.1	5.15 ± 1.99	84.2 ± 27.0	0.0034
FEF_50%_ (L/s)	2.04 ± 1.02	47.3 ± 23.6	3.20 ± 1.63	72.4 ± 31.0	<0.0001
FEF_75%_ (L/s)	0.81 ± 0.49	43.4 ± 28.0	1.22 ± 0.74	63.6 ± 36.3	<0.0001
Angle *β*	172 ± 21		188 ± 20		<0.0001
SF_25%_	0.62 ± 0.34		0.19 ± 0.07		<0.0001
SF_50%_	0.61 ± 0.19		0.25 ± 0.13		<0.0001

Each value is shown as the mean ± SD.

**Table 2:** Correlation between % changes of angle *β*, SF_25%_, and SF_50%_ and % changes of spirometric parameters after SABA inhalation.

	Angle *β* versus		SF_25%_ versus		SF_50%_ versus	
	*r*	*P*	*r*	*P*	*r*	*P*
FVC (% change)	0.0043	0.98	−0.085	0.57	−0.32	0.11
FEV_1_ (% change)	0.32	0.1	−0.23	0.24	−0.32	0.1
PEF (% change)	−0.15	0.47	0.096	0.63	0.0067	0.97
FEF_25–75%_ (% change)	0.57	**0.0017**	−0.43	**0.024**	−0.31	0.12
FEF_25%_ (% change)	0.075	0.71	−0.12	0.54	−0.35	0.074
FEF_50%_ (% change)	0.71	**<0.0001**	−0.35	0.078	−0.25	0.21
FEF_75%_ (% change)	0.47	**0.013**	−0.53	**0.0046**	−0.54	**0.0041**
SF_50%_ (% change)	0.12	0.54	−0.9	**<0.0001**	—	—
SF_25%_ (% change)	0.33	0.089	—	—	—	—
Angle *β* (% change)	—	—	—	—	—	—

Bold text indicates significant correlation between the parameters.
